# Are Raw BIA Variables Useful for Predicting Resting Energy Expenditure in Adults with Obesity?

**DOI:** 10.3390/nu11020216

**Published:** 2019-01-22

**Authors:** Maurizio Marra, Iolanda Cioffi, Rosa Sammarco, Lidia Santarpia, Franco Contaldo, Luca Scalfi, Fabrizio Pasanisi

**Affiliations:** 1 Department of Clinical Medicine and Surgery, Federico II University Hospital, Pansini 5, 80131 Naples, Italy; marra@unina.it (M.M.); rosa.sammarco@unina.it (R.S.); lidia.santarpia@unina.it (L.S.); 2 Interuniversity Centre for Obesity and Eating Disorders, Federico II University Hospital, Pansini 5, 80131 Naples, Italy; contaldo@unina.it (F.C.); pasanisi@unina.it (F.P.); 3 Department of Public Health, Federico II University, Pansini 5, 80131 Naples, Italy; scalfi@unina.it

**Keywords:** obesity, basal metabolic rate, body composition, phase angle

## Abstract

This study aimed to develop and validate new predictive equations for resting energy expenditure (REE) in a large sample of subjects with obesity also considering raw variables from bioimpedance-analysis (BIA). A total of 2225 consecutive obese outpatients were recruited and randomly assigned to calibration (*n* = 1680) and validation (*n* = 545) groups. Subjects were also split into three subgroups according to their body mass index (BMI). The new predictive equations were generated using two models: Model 1 with age, weight, height, and BMI as predictors, and Model 2 in which raw BIA variables (bioimpedance-index and phase angle) were added. Our results showed that REE was directly correlated with all anthropometric and raw-BIA variables, while the correlation with age was inverse. All the new predictive equations were effective in estimating REE in both sexes and in the different BMI subgroups. Accuracy at the individual level was high for specific group-equation especially in subjects with BMI > 50 kg/m^2^. Therefore, new equations based on raw-BIA variables were as accurate as those based on anthropometry. Equations developed for BMI categories did not substantially improve REE prediction, except for subjects with a BMI > 50 kg/m^2^. Further studies are required to verify the application of those formulas and the role of raw-BIA variables for predicting REE.

## 1. Introduction

An accurate prediction of resting energy expenditure (REE) is crucial for assessing energy requirements in individuals with obesity, since REE accounts for 60–70% of total energy needs in sedentary people [1,2]. Indirect calorimetry (IC) is viewed as the criterion method for REE measurement, but it is time-expensive and frequently not available in clinical setting. Alternatively, REE is frequently estimated by predictive equations based on easily available variables such as age, height, body weight, etc.

Generally, predictive equations for REE provide acceptable results when applied in the population from which they are derived. To date, several predictive equations for REE have been developed for subjects with obesity, resulting not always suitable for predicting REE as far as those obtained in the general population [3,4,5,6]. These shortcomings might be related to the heterogeneity of study populations, methodological drawbacks, and REE variability [7]. Based on a recent systematic review by Madden et al. [6], no single equation provided accurate estimates of REE in adults with obesity, suggesting that accuracy prediction varied across BMI subgroups. Available results from our group [4] and others [3,8], showed that specific equations for obesity proposed by Muller [7] and Huang [9] might be acceptable for estimating REE at the population level when BMI is higher than 40 kg/m^2^; while Madden et al. [6] recommended the Mifflin equations. However, the precision of prediction accuracy decreases with increasing BMI (>50 kg/m^2^), with underestimated values in approximately 50% of subjects [3,4].

It is commonly accepted that fat-free mass (FFM) is the major determinant of REE in normal-weight subjects, including both highly and moderately metabolically active tissues and organs, such as brain, liver, heart, kidney, and muscle. Specifically, in subjects with obesity, the increase in body weight may not strictly reflect changes in FFM due to an excessive presence of fat mass (FM) [10,11,12], which is less metabolically active, leading to larger errors when estimating REE [13].

Hence, it is still uncertain to which extent the use of body composition (BC) variables could effectively enhance REE prediction, since data are still controversial [4,14,15,16,17]. From a practical point of view, bioimpedance analysis (BIA) is the most commonly used tool for BC assessment in the general population [18]. However, in subjects with obesity, the interpretation of BIA results is highly dependent on the equation used to estimate FFM and FM [19,20]. As an alternative, raw BIA data might be taken into consideration. A relationship between energy expenditure and raw BIA variables such as bioimpedance index (BI-index) and phase angle (PhA) is expected since BI-index is a direct, accurate proxy of FFM, while PhA is related to body cell mass [21].

Based on this background, the objectives of the present study were: (1) To develop and validate new predictive equations for REE specific for subjects with obesity, especially those with a BMI > 40 kg/m^2^; (2) to assess whether including raw BIA variables into the model enhances the prediction power of the regression; and (3) to establish if specific-BMI equations could improve the reliability of prediction.

## 2. Materials and Methods

### 2.1. Study Population

The present study was a retrospective analysis of data collected between 2005 and 2017 from consecutively adult outpatients with obesity undergoing routine procedures to evaluate nutritional status (including biochemistry, BIA and indirect calorimetry) at the Internal Medicine and Clinical Nutrition Unit of the Federico II University Hospital in Naples, Italy. The study was carried out in accordance with the Helsinki Declaration and received the approval of the Ethical Committee of Federico II University. Informed consent was obtained from all patients. 

A total of 2225 Italian caucasian patients with obesity, 1428 females and 797 males were selected, according to the following inclusion criteria: Both sexes, age 18–65 years and BMI ≥ 30 kg/m^2^. Exclusion criteria were as follows: current participation in a weight loss program, presence of active inflammatory diseases, dysthyroidism, type 2 diabetes mellitus, pregnancy, lactation, or daily use of prescription medication or drugs known to influence energy metabolism. 

All measurements were performed early in the morning after a fasting period of 8–10 h according to standardized conditions, i.e., abstention from alcohol, smoking, and vigorous physical activity for 24 h prior to the assessment. According to the protocol, smoking was not allowed for occasional and current smokers on the morning of the test, until the end of measurements; however, current smokers were asked to maintain their smoking habits on the day before [22]. Data were excluded from analysis if the respiratory quotient was outside the expected range (0.71–0.90), when measured REE was ±3 standard deviations outside the mean REE, or if the presence of overt peripheral edema was detected.

### 2.2. Anthropometry and Bioelectrical Impedance Analysis

Body weight and height were measured to the nearest 0.1 kg and 0.5 cm, respectively. Measurements were taken while the subject wore light clothes and no shoes using a platform beam scale with a built-in stadiometer (Seca 709, Seca, Hamburg, Germany). BMI was calculated as body weight expressed in kilograms divided by squared height reported in meters. The coefficient of variation on three consecutive measures (in 20 subjects) was always <0.1% for body weight and <0.3% for height. 

BIA [23] was performed at 50 kHz (Human In Plus II, DS Medica, Milan, Italy) at room temperature (22–25 °C). Measurements were carried out on the nondominant side of the body, in the post-absorptive state, after voiding and with the subject in the supine position for 20 min. The BIA variables considered (data produced by the device) were resistance (R), reactance (Xc), and phase angle (PhA). Bioimpedance index (BI-index) was calculated as the ratio height^2^/resistance (cm^2^/Ω). The coefficient of variation on three consecutive measures (20 subjects) was always <2% for R and < 4% for PhA.

### 2.3. Indirect Calorimetry

Resting Energy Expenditure (REE) was measured by indirect calorimetry [24] using a canopy system (V max29, Sensor Medics, Anaheim, CA, USA). This is a well-known device used worldwide for assessing energy expenditure. The instrument was routinely checked by burning ethanol, while oxygen and carbon dioxide analyzers were calibrated on the test day using nitrogen and standardized gases (mixtures of nitrogen, carbon dioxide and oxygen). 

Measurement conditions for IC were defined following the suggestions made by Compher et al. [25] and Fullmer et al. [26]. REE was assessed at an ambient temperature of 22–25 °C and, in fertile women, during the follicular phase to avoid any potential effects of the menstrual cycle. Subjects lay down on a bed in a quiet environment for a 15-min adaptation period. Afterward, oxygen consumption and carbon dioxide production were measured for 45 min, discarding the first 5 min. The interday coefficient of variation (as determined in six individuals with obesity on consecutive days) was less than 3%. Energy expenditure was calculated using the abbreviated Weir’s formula, neglecting protein oxidation [27]. 

### 2.4. Statistical Analysis

Statistical analyses were performed using IBM SPSS (Version 24.0, IBM Corp, Armonk, NY, USA) for males and females separately (sex-specific whole sample groups). Data are presented as mean ± standard deviation (SD), and statistical significance was defined as *p* < 0.05.

Within each sex, patients were also split into: BMI subgroup 1 = 30–39.9 kg/m^2^; BMI subgroup 2 = 40–49.9 kg/m^2^; BMI subgroup 3 ≥ 50 kg/m^2^. In addition, as outlined in Table 1 and Table 2 subjects were randomly assigned to a calibration or a validation subset in a way that the ratio between them remained constant in whole sample groups and BMI subgroups. As far as statistical power is concerned, it may be mentioned that, for an alpha level of 0.05 and a power of 0.80, the sample size requested for the association between variables is *n* = 783 for *r* = 0.100, *n* = 198 for *r* = 0.200, and *n* = 85 for *r* = 0.300.

The Kolmogorov-Smirnov Test and the Shapiro-Wilk Test were used as tests of normality, which is to examine if variables were normally distributed. Data were compared between sexes using one-way ANOVA, while linear correlation was applied for evaluating associations between variables. Multivariate linear regression analysis was performed to develop the new predictive equations, with measured REE bi IC (MREE) as dependent variable. We generated sex-specific models as follows: in Model 1, age, weight, height, and BMI were set as predictors, while in Model 2, we added to the model the raw BIA variables BI-index and PhA. Coefficient of determination (*R*^2^) and standard error of the estimate (SEE) were considered for assessing the predictive power of formulas.

A linear regression analysis was used, considering that this is the model by far more frequently used for the prediction of REE in order to offer simple formulas to be applied in the clinical setting. The regression equations derived from the calibration subsets were applied to the validation subsets. Bias, i.e., the average percent difference between predicted REE (PREE) and MREE, was used as a measure of accuracy at the group level and found acceptable if within ± 5% [28,29]. Concurrently, the percentage of patients with a PREE within 90–110% of MREE was used as a measure of accuracy at the individual level; values lower than 90% were classified as under prediction and values higher than 110% as over prediction. Finally, comparisons of PREE-MREE differences vs. mean PREE-MREE values were performed by Bland-Altman plots to estimate the limits of agreement [30].

Finally, PREE values of these new formulas were compared to those of other equations as the Harris-Benedict [31], Henry [32], Mifflin [33], Muller [7], and Lazzer equations [34]. 

## 3. Results

As already mentioned, sex-specific whole sample groups and BMI subgroups were randomly split into a calibration subset and a validation subset (*n* = 1680 and 545, respectively). Anthropometric, raw BIA variables, and MREE data are summarized for the calibration and validation groups in Table 1 and Table 2, respectively.

Pearson correlation coefficients for the association of MREE with individual characteristics or raw BIA variables are shown in Table 3. In the whole sample groups MREE was directly correlated with all anthropometric variables, whereas age displayed an inverse effect. In addition, there was a moderate association of MREE with BI-index and PhA in both sexes. Overall, body weight showed the strongest correlation with MREE in both genders in the whole sample groups as well as BMI subgroups. Only in Group 3 (BMI > 50 kg/m^2^) did BI-index emerge as a better determinant of REE than body weight. 

As a further step, multiple regression analysis was performed to assess the relationships between MREE and different combinations of potential predictors. Age and basic anthropometric characteristics (height, weight, and BMI) were considered first (Model 1) to develop the following equations:

Males: REE = 13.3 × Weight − 2.54 × Age + 866 (unstandardized regression coefficients, *R*^2^ = 0.621; SEE = 259 kcal) (1)

Females: REE = 13.5 × Weight − 2.40 × Age + 584 (unstandardized regression coefficients, *R*^2^ = 0.688; SEE = 208 kcal) (2)

In both males and females, the standardized regression coefficients (*β*) were much higher for body weight (*β* = 0.775 and *β* = 0.832, respectively) compared to age (*β* = −0.070 and *β* = −0.089). Height entered into the model only in females (un-standardized regression coefficient −2.43), leading to a very small decrease in SEE values, while BMI did not emerge as a significant predictor in either sex. 

Next, the combination of individual characteristics and raw BIA variables was considered (Model 2), leading to the following equations:

Males: REE=11.5 × Weight − 3.32 × Age + 6.15 × BI-index + 46.1 × PhA + 313 (unstandardized regression coefficients, *R*^2^ = 0.647; SEE = 250 kcal) (3)

Females: REE=12.3 × Weight − 2.10 × Age + 4.96 × BI-index + 42.7 × PhA + 143 (unstandardized regression coefficients, *R*^2^ = 0.707; SEE = 201 kcal) (4)

Compared to Equations (1) and (2) (Model 1), in both sexes, the inclusion of raw-BIA variables (Equations (3) and (4)) determined an increase in *R*^2^ and a small decrease in SEE. While MREE was significantly related to the BI-index (*β* = 0.153 in males and *β* = 0.162 in females), the main determinant was still body weight (*β* = 0.668 in males and *β* = 0.727 in females). 

Predictive equations derived in BMI subgroups are shown in Table 4. In Model 1, body weight was the main predictor of MREE, whereas age was much less relevant. When BI-index and PhA were included in the model, body weight remained a significant predictor of MREE in both BMI subgroups 1 and 2, but not in subjects with a BMI > 50 kg/m^2^. In this latter group, BI-index emerged as the best determinant of MREE (*β* = 0.332 in males and *β* = 0.317 in females); thus, model 2 improved *R*^2^ and decreased SEE values of approximately 15 kcal/d in males and females compared to Model 1. 

### Validation of the New Predictive Equations

To evaluate the accuracy of the new predictive equations in the whole sample as well as each BMI subgroup, 545 individuals with obesity (195 males and 350 females) were assigned to validation groups. Prediction accuracy at the group level, assessed by the difference between PREE and MREE (bias), is reported in Table 5.

**Figure 1:**
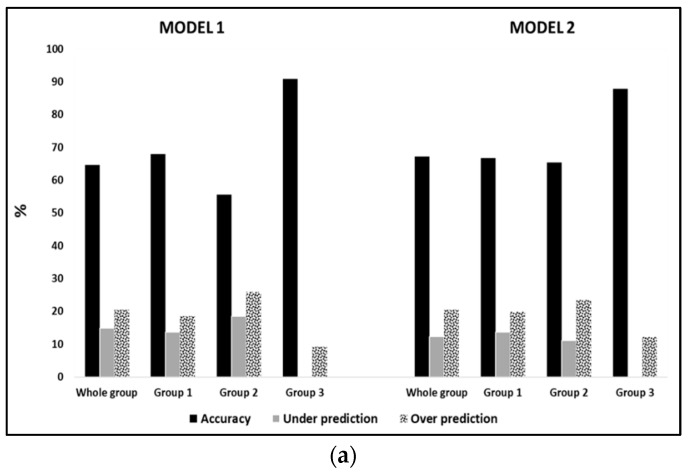

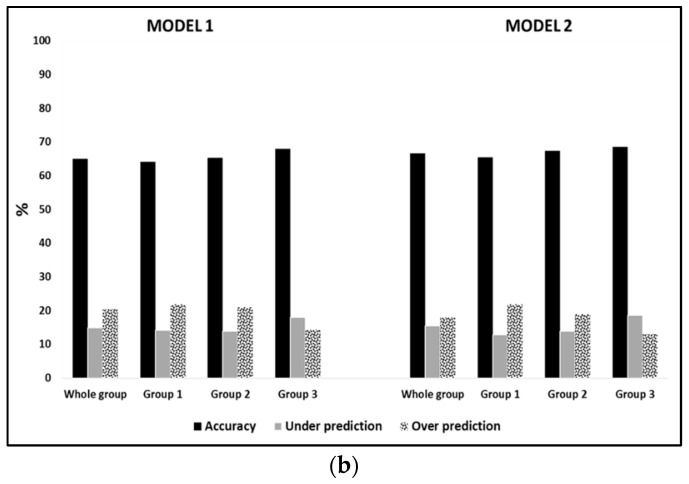
Prediction accuracy for REE measurements within ± 10% using the new predictive equations (model 1 and 2, Equations (1)–(4)) in 153 males (**a**) and 350 females (**b**) with obesity as well as in the BMI subgroups (Group 1 = 30–39.9 kg/m^2^; Group 2 = 40–49.9 kg/m^2^; Group 3 > 50 kg/m^2^).

All new developed predictive equations were accurate at the group level for either the whole sample groups or each BMI subgroup, since mean bias was always within the range ± 3%. As an index of accuracy at the individual level, the percentage of subjects with a PREE within ± 10% of MREE is shown in Figure 1. In the whole sample validation groups, values (Equations (1)–(4)) were around 65%, while as far as BMI subgroups were concerned, we observed the highest value in males with a BMI > 50 kg/m^2^.

When REE was predicted using selected equations from the literature, we found that the bias was acceptable for the Harris-Benedict equation only (M = −4.4%, F = −3.4%), and more negative for the Henry (M = −6.1%, F = −8.2%), Mifflin (M = −5.8%, F = −6,4%), Muller (M = −9.5%, F = −10.1%), and Lazzer equations (M = −5.3%, F = −6.2%). Overall, accuracy at the individual level was low (<57%) in both sexes and for all the equations considered.

**Figure 2:**
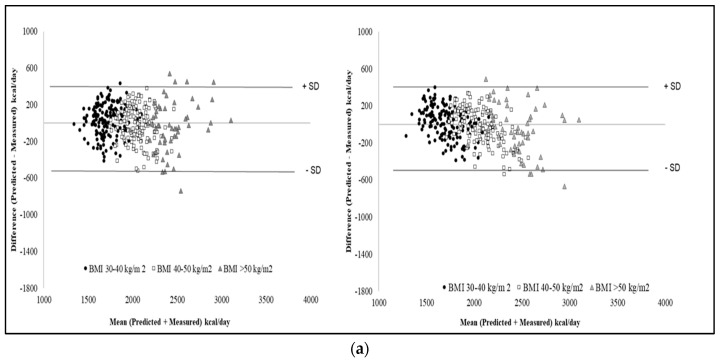

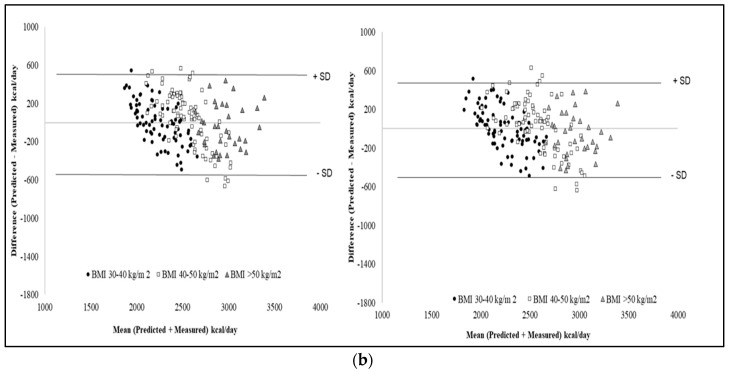
Bland-Altman plots between differences and mean predicted-measured REE using the new predictive equations in 195 males (**a**: Equations (1) and (3)) and 350 (**b**: Equations (2) and (4)) females with obesity. The dotted lines represent 2 SDs from the mean (limits of agreement).

Finally, the Bland-Altman plots of PREE-MREE differences vs mean PREE-MREE values were shown in Figure 2 for Equations (1)–(3) (males) and (2)–(4) (females), since those selected plots highlight the best agreement.

## 4. Discussion

The aim of this study was to investigate the relationships of REE with age, main anthropometric and raw BIA variables, and to develop new predictive equations for REE in individuals with moderate or severe obesity. Our results showed that REE is largely determined by body weight, at least if the BMI is lower than 50 kg/m^2^, whereas an inverse correlation emerged with age. Interestingly, when the BMI exceeds 50 kg/m^2^, the BI-index is the best determinant of REE in both sexes. Finally, we found that prediction accuracy was good for all equations, resulting significantly higher in males with BMI above 50 kg/m^2^. However, from a clinical point of view, the inclusion of raw BIA variables in the equation model did not significantly improve the prediction power of equations. 

The accuracy of predictive equations is expected to be higher when they are applied in individuals sharing the same characteristics. For instance, equations developed in the general population should not be applied in individuals with severe obesity, unless specifically validated. Previously, we have assessed prediction accuracy in our outpatients, reporting that only the Muller [7] equations were suitable for predicting REE (PREE-MREE difference lower than 5%); however, none of the equations reported an accurate prediction at the individual level, with poorer results in subjects with BMI > 40 kg/m^2^ [4], suggesting the need of new predictive equations which are specific for different classes of BMI and also concerning subjects with severe obesity. In fact, to our knowledge, only a few studies, with small sample sizes, developed equations for class III obesity, i.e., BMI > 40 kg/m^2^ [34,35].

In the present study, we developed new predictive sex-specific equations, based on basic individual parameters alone (age, weight, height, and BMI) (Model 1) or in combination with raw BIA variables (BI-index and PhA) (Model 2). Our new predictive equations showed similar SEE values between males and females in the calibration groups. Body weight resulted the best predictor of REE in both sexes. On the other side, we found an inverse association between REE and age in all predictive models, as previously reported [36]; however, the age contribution to REE prediction was small (~3 kcal).

Beyond the use of BIA-derived FFM and FM, we investigated whether and to what extent the addition of raw BIA variables, PhA and BI-index, may improve REE prediction in subjects with obesity. Although raw data can be device-dependent, values for Z, R and Xc (PhA is directly given or calculated from R and Xc), measured at 50 kHz, are usually made available by almost all scientific BIA devices (single or multifrequency). Raw BIA variables have gained considerable attention in recent years because they are not only associated with FFM, but they are also markers of extracellular/intracellular distribution of water, BMC, integrity and muscle quality [37], without the assumptions made for estimating body compartments [20]. Thus, they are expected to be potential predictors of REE. Additionally, it has been reported that PhA may be a predictor of morbidity and mortality [28]. Thus, we firstly assessed predictive equations of REE including raw BIA variables alone (males: *R*^2^ = 0.343, SEE = 341 kcal; females: *R*^2^ = 0.439, SEE = 279 kcal) or in combination with age (males: *R*^2^ = 0.390, SEE = 329 kcal; females: *R*^2^ = 0.459, SEE = 274 kcal). The prediction power of these equations is much lower compared to those including basic individual characteristic (Model 1). Secondly, when we included, in the model, both individual characteristic and raw BIA variables, we observed an increase of *R*^2^ and a decrease in SEE values in both sexes. 

In the validation group, accuracy at the population level was acceptable since it ranged within ± 3%, with similar bias using either Model 1 or Model 2. Likewise, accuracy at individual level was higher (~65%) than that observed in previous studies [3,4,34,38]. These results were in accordance with previous works [7,9,29,34,39] that developed predictive equations in subjects with obesity, including BIA-derived FFM and FM. According to a recent paper [17], the use of BIA-derived FFM may lead to a small improvement in accuracy prediction. Similarly, our raw BIA-based equations showed a slight improvement in prediction accuracy at both population and individual level compared to the weight-based equations (Table 5 and Figure 1). Indeed, from a physiological point of view, it is worth noting the relationship between REE and PhA, which is considered as an index of BCM. With regard to BI-index, this variable is similarly included together with (not instead of) body weight also in the BIA equation used to predict FFM. Finally, when these new equations were compared with others previously published, we found that bias at the population level was within ± 5% for only the Harris-Benedict equations, while accuracy at the individual level was much lower (~56.5%) than for those proposed in the present paper (~65%).

Finally, since the number of people with an extreme BMI (>50 kg/m^2^) is continuously increasing, it is crucial to provide a reliable tool for assessing REE in planning adequate nutritional advice for weight management and bariatric surgery procedures. According to the literature, most studies reported that the accuracy of prediction decreased considerably with an increasing BMI (>40 kg/m^2^) [3,4]; hence, we split the study population into three BMI subgroups (subgroup 1 = 30–39.9 kg/m^2^; subgroup 2 = 40–49.9 kg/m^2^; and subgroup 3 > 50 kg/m^2^). Body weight was the major predictor of REE, as previously observed in the whole study sample; however, in Group 3, we found that BI-index was a slightly stronger predictor than body weight.

In the validation samples, prediction accuracy at the group level was comparable between BMI-specific equations and those developed for the whole study sample, as shown in Table 5. Compared to previous results [4], accuracy at individual level was higher (>65%) in all BMI subgroups and much higher comparing to the equations from the literature. Surprisingly, prediction accuracy was very high in subjects with BMI > 50 kg/m^2^ (~85% in males; ~67% in females).

This is, to our knowledge, the first study that specifically develops and cross-validates predictive equations for subjects with severe obesity and opts for using raw BIA variables to estimate REE in individuals with BMI > 50 kg/m^2^. According to the literature, no previous work has developed predictive formulas for different classes of obesity. Muller et al. [7] generated equations according to BMI categories, without including subjects with BMI ≥ 40 kg/m^2^; while Lazzer et al. [34] described the individual characteristics of the study population according to BMI subgroups (40–45 kg/m^2^; 45–50 kg/m^2^ and > 50 kg/m^2^) without formulating specific-BMI equations. Furthermore, none of studies published so far used raw BIA variables to predict REE in subjects with obesity. This new approach could be helpful to avoid issues related to different equations for BC measurement that can modify the prediction of the formulas and alter their validity. 

Overall, we performed a cross-sectional protocol in a large sample of individuals with obesity using known and documented methods and in line with previous studies that have derived predictive equations for REE from healthy subjects as well as from patients with different diseases. However, some limitations need to be considered. Firstly, this is a single-center study including adult outpatients, therefore our findings need to be substantiated in other subgroups or in different clinical settings. Secondly, due to the aim of the study and inclusion/exclusion criteria, the effects of comorbidities cannot be evaluated from our data and no information can be provided on elderly subjects with obesity. Then, unfortunately, no data were available on BC by other techniques such as DXA. Last, but not least, other proxy measures of obesity and/or fat distribution need to be considered in the future, being widely-available variables, for improving the predictive power of the formulas. 

## 5. Conclusions

In conclusion, this study showed that new predictive equations were accurate at the population level; although, the inclusion of raw BIA variables did not substantially enhance the prediction accuracy from a practical point of view in clinical settings. Regarding the use of predictive equations based on BMI categories, our results highlighted that these equations were not strictly necessary for REE prediction in subjects with mild/moderate obesity but can be useful in those with a BMI higher than 50 kg/m^2^. Finally, accuracy at the individual level was higher compared to those assessed by previous studies, especially in subject with severe obesity; nevertheless, we still recognized the importance of measuring REE by IC. Further studies are necessary to verify the application of these equations, especially in the group of subjects with severe obesity, as well as to investigate the role of raw BIA variables in predicting REE.

## Figures and Tables

**Table 1:** Characteristics of BMI subgroups and the entire study sample for the calibration groups.

		BMI Subgroup 1		BMI Subgroup 2		BMI Subgroup 3		All Subjects	
		M (*n* = 251)	F (*n* = 483)	M (*n* = 248)	F (*n* = 424)	M (*n* = 103)	F (*n* = 171)	M (*n* = 602)	F (*n* = 1078)
**Age**	years	35.4 ± 12.1	35.4 ± 13.5	33.4 ± 11.8	34.4 ± 12.3	33.2 ± 10.8 *	36.9 ± 11.1	34.2 ± 11.8	35.2 ± 12.7
**Weight**	kg	109 ± 11 *	91 ± 10	135 ± 13 *	116 ± 12	166 ± 17 *	144 ± 14	129 ± 24 *	109 ± 22
**Height**	cm	175 ± 6 *	161 ± 6	174 ± 6 *	162 ± 6	173 ± 8 *	161 ± 6	174 ± 7 *	161 ± 6
**BMI**	kg/m^2^	35.5 ± 2.9 *	35.0 ± 2.9	44.4 ± 2.8	44.4 ± 2.8	55.3 ± 4.2	55.8 ± 4.8	42.6 ± 7.7	42.0 ± 8.1
**BI-Index**	cm^2^/Ω	73.1 ± 9.5 *	51.6 ± 6.7	79.1 ± 10.7 *	58.2 ± 7.5	88.2 ± 12.3 *	65.8 ± 9.1	78.1 ± 11.8 *	56.5 ± 9.0
**PhA**	degrees	7.33 ± 0.89 *	6.58 ± 0.82	7.36 ± 0.97 *	6.73 ± 0.83	7.35 ± 1.03 *	6.62 ± 0.98	7.34 ± 0.95 *	6.64 ± 0.85
**MREE**	kcal/d	2225 ± 307 *	1698 ± 221	2577 ± 344 *	2112 ± 263	3009 ± 241 *	2433 ± 299	2504 ± 420 *	1978 ± 372
**RQ**		0.845 ± 0.053	0.843 ± 0.051	0.840 ± 0.060	0.837 ± 0.061	0.842 ± 0.062	0.829 ± 0.062	0.842 ± 0.061	0.839 ± 0.064

* *p* < 0.05 ANOVA between sexes. Data are expressed as mean ± SD. Subgroup 1: BMI = 30–39.9 kg/m^2^; Subgroup 2: BMI = 40–49.9 kg/m^2^; Subgroup 3: BMI > 50 kg/m^2^; d = day; M = male; F = female; BI-index = bioimpedance index; PhA = phase angle; MREE = measured resting energy expenditure; RQ = respiratory quotient; and Ω = ohm.

**Table 2:** Characteristics of BMI subgroups and the entire study sample for the validation group.

		BMI Subgroup 1		BMI Subgroup 2		BMI Subgroup 3		All Subjects	
		M (*n* = 81)	F (*n* = 156)	M (*n* = 81)	F (*n* = 138)	M (*n* = 33)	F (*n* = 56)	M (*n* = 195)	F (*n* = 350)
**Age**	years	34.2 ± 11.3	34.9 ± 13.4	34.1 ± 12.1	34.5 ± 12.2	34.1 ± 12.0 *	36.7 ± 11.4	34.1 ± 11.7	35.1 ± 12.7
**Weight**	kg	109 ± 11.4 *	90 ± 10	135 ± 12 *	115 ± 10	162 ± 16 *	143 ± 15	129 ± 23 *	108 ± 22
**Height**	cm	176 ± 6 *	161 ± 5	175 ± 6 *	162 ± 6	173 ± 7 *	161 ± 6	175 ± 7 *	161 ± 6
**BMI**	kg/m^2^	35.3 ± 3.0 *	34.6 ± 2.8	44.2 ± 3.1	43.9 ± 2.6	55.3 ± 4.5	55.3 ± 4.5	42.2 ± 7.4	41.6 ± 8.0
**BI-Index**	cm^2^/Ω	73.3 ± 9.6 *	51.2 ± 6.7	79.7 ± 10.4 *	57.8 ± 6.5	87.8 ± 11.2 *	65.4 ± 10.6	78.4 ± 11.4 *	56.1 ± 9.0
**PhA**	degrees	7.36 ± 0.86 *	6.56 ± 0.83	7.39 ± 0.89 *	6.71 ± 0.92	7.28 ± 1.00 *	6.57 ± 1.03	7.36 ± 0.89 *	6.62 ± 0.9
**MREE**	kcal/d	2240 ± 290 *	1683 ± 213	2545 ± 346 *	2086 ± 260	2979 ± 217 *	2480 ± 299	2492 ± 400 *	1970 ± 381
**RQ**		0.838 ± 0.051	0.846 ± 0.052	0.826 ± 0.053	0.836 ± 0.062	0.829 ± 0.047	0.823 ± 0.061	0.831 ± 0.053	0.838 ± 0.054

* *p* < 0.05 ANOVA between sexes. Data are expressed as mean ± SD. Subgroup 1: BMI = 30–39.9 kg/m^2^; Subgroup 2: BMI = 40–49.9 kg/m^2^; Subgroup 3: BMI > 50 kg/m^2^; d = day; M = male; F = female; BI-I = bioimpedance index; PhA = phase angle; MREE = measured resting energy expenditure; RQ = respiratory quotient; and Ω = ohm.

**Table 3:** Linear correlation between REE, individual characteristics and BIA parameters according to sex and BMI.

	BMI Subgroup 1		BMI Subgroup 2		BMI Subgroup 3		All Subjects	
	M (*n* = 251)	F (*n* = 483)	M (*n* = 248)	F (*n* = 424)	M (*n* = 103)	F (*n* = 171)	M (*n* = 602)	F (*n* = 1078)
**Age**	−0.211 ***	−0.254 ***	−0.134 *	−0.238 ***	0.07	−0.023	−0.160 ***	−0.126 ***
**Weight**	0.638 ***	0.569 ***	0.569 ***	0.629 ***	0.458 ***	0.462 ***	0.785 ***	0.826 ***
**Height**	0.476 ***	0.375 ***	0.400 ***	0.498 ***	0.356 ***	0.235 ***	0.252 ***	0.267 ***
**BMI**	0.402 ***	0.426 ***	0.350 ***	0.371 ***	0.221 ***	0.334 ***	0.710 ***	0.770 ***
**BI-index**	0.384 ***	0.424 ***	0.418 ***	0.416 ***	0.471 ***	0.486 ***	0.572 ***	0.652 ***
**Phase angle**	0.115 *	0.150 **	0.175 ***	0.086 *	−0.072	0.204 **	0.093 **	0.123 ***

* *p* < 0.05; ** *p* < 0.01; *** *p* < 0.001. Group 1: BMI = 30–39.9 kg/m^2^; Group 2: BMI = 40–49.9 kg/m^2^; Group 3: BMI > 50 kg/m^2^; M = male; F = female; and BI-index = Bioimpedance index.

**Table 4:** New predictive equations based on model 1 or 2 in each BMI subgroups.

Model	Sex	Predictive Equations	*R* ^2^	*SEE (kcal/d)*
**BMI Subgroup 1** (*n* = 251 M; *n* = 483 F)				
1	M	REE = 16.6 × Weight − 3.23 × Age + 536	0.423	233
2	M	REE = 14.6 × Weight − 3.32 × Age + 34.9 × PhA + 4.7 × BI-I + 157	0.444	230
1	F	REE = 11.9 × Weight − 2.48 × Age + 704	0.347	178
2	F	REE = 10 × Weight − 2.34 × Age + 32 × PhA + 4.7 × BI-I + 416	0.374	175
**BMI Subgroup 2** (*n* = 248 M; *n* = 424 F)				
1	M	REE=15.4 × Weight – 2.71 × Age + 585	0.333	282
2	M	REE=12.1 × Weight − 3.48 × Age + 52.1 × PhA + 6.12 × BI-I + 191	0.373	274
1	F	REE=13.8 × Weight − 2.67 × Age + 597	0.408	202
2	F	REE= 12.6 × Weight − 2.64 × Age + 31.2 × PhA + 3.4 × BI-I + 324	0.422	200
**BMI Subgroup 3** (*n* = 103 M; *n* = 171 F)				
1	M	REE = 6.32 × Weight + 1960	0.210	215
2	M	REE = 4.09 × Weight + 6.81 × BI-I + 1730	0.315	200
1	F	REE = 9.59 × Weight +1051	0.213	261
2	F	REE = 6.90 × Weight + 88.6 × PhA + 11.4 × BI-I + 84	0.324	245

M = males; F = females; unstandardized regression coefficients. Group 1: BMI = 30–39.9 kg/m^2^; Group 2: BMI = 40–49.9 kg/m^2^; Group 3: BMI > 50 kg/m^2^; d = day; REE = resting energy expenditure; BI-I = bioimpedance index; PhA = phase angle; SEE = standard error of the estimate.

**Table 5:** Bias (mean percentage error between predicted and measured REE) for the new predictive equations in the entire study groups as well as BMI subgroups.

	Bias (Mean Percentage Difference between Predicted and Measured REE)															
	All Subjects				BMI Subgroup 1				BMI Subgroup 2				BMI Subgroup 3			
	M (*n* = 195)		F (*n* = 350)		M (*n* = 81)		F (*n* = 156)		M (*n* = 81)		F (*n* = 138)		M (*n* = 33)		F (*n* = 56)	
Model	1	2	1	2	1	2	1	2	1	2	1	2	1	2	1	2
**Eq developed in:**															
**Whole sample**	1.5	1.4	1.1	0.8	0.9	1.4	3.0	2.7	2.5	2.6	−0.7	−0.5	−1.8	−1.2	−1.2	−1.2
**Subgroup 1**					1.1	1.0	1.5	1.2								
**Subgroup 2**									2.2	2.0	1.2	0.9				
**Subgroup 3**													0.5	0.4	−1.2	−0.6

Subgroup 1: BMI = 30–39.9 kg/m^2^; subgroup 2: BMI = 40–49.9 kg/m^2^; subgroup 3: BMI > 50 kg/m^2^; Eq = equation; M = males; F = females.
